# Rural retention strategies in the South-East Asia Region: evidence to guide effective implementation

**DOI:** 10.2471/BLT.19.245662

**Published:** 2020-10-05

**Authors:** Tomas Zapata, James Buchan, Viroj Tangcharoensathien, Andreasta Meliala, Indika Karunathilake, Nilar Tin, Sulakshana Nandi, Tashi Tobgay, Thinakorn Noree

**Affiliations:** a Health Systems Development Department, World Health Organization, South-East Asia Region, Metropolitan Hotel Office Block, Bangla Sahib Road, Gole Market, Sector 4, 110001, New Delhi, India.; b World Health Organization Collaborating Centre, University of Technology, Sydney, Australia.; c International Health Policy Program, Bangkok, Thailand.; d Faculty of Medicine, Public Health and Nursing, Universitas Gadjah Mada, Yogyakarta, Indonesia.; e World Health Organization Collaborating Centre for Medical Education, University of Colombo, Colombo, Sri Lanka.; f Ministry of Health and Sport, Nay Pyi Taw, Myanmar.; g Public Health Resource Network, Chhattisgarh, India.; h Institute of Health Partners, Thimphu, Bhutan.

The availability of a functioning primary health-care system for populations in rural and underserved areas is crucial for reducing morbidity and mortality, and improving health indicators. A fully functioning primary health-care system is also a precondition to achieving universal health coverage (UHC).^1^

The health workforce is the backbone of primary health-care systems.^2^ Sufficient, well trained, competent and motivated primary health-care workers in rural and underserved areas are particularly important to provide quality primary health-care services and enhance equity as these services become accessible to more people.^3^^,^^4^

However, many countries across the world experience challenges in recruiting and retaining health-care workers where they are most needed, that is, in rural and underserved areas.^5^ Workforce shortages, geographical maldistribution, suboptimal skill mix, low motivation and high absenteeism contribute to poor primary health-care performance in rural and underserved areas.^4^^,^^5^ Improving rural retention of the health workforce is vital to the functioning of primary health care – and therefore to achieving UHC.^3^

The protracted challenges in rural recruitment and retention of health-care workers are not new. In 2010, the World Health Organization (WHO) launched the guidelines *Increasing access to health workers in remote and rural areas through improved retention: global policy recommendations.* The guidelines recommend 16 policy interventions grouped in four main categories of intervention: education; regulation; financial incentives; and professional and personal support.^4^ While the framework has been widely used to design and implement policy interventions by countries, its monitoring framework and indicators have not been widely applied to monitor progress and assess the impact of policy interventions.^5^ Other public health actors have proposed alternative monitoring frameworks, but policy-makers have not widely adopted them.^6^

The 2010 WHO guidelines on rural retention are in the process of being updated, including a new evidence review and an effectiveness assessment of the recommendations; the guidelines should be published in late 2020.

## Rural retention in South-East Asia

All Member States in the South-East Asia Region, except for Democratic People's Republic of Korea and Maldives, report health workforce shortages as measured by WHO’s benchmark of 44.5 professional staff per 10 000 population. Maldistribution of health-care workers in rural areas,^7^ where 66% of the 2 billion population of South-East Asia live, is a common challenge across all countries in this region.^8^ To mitigate these challenges, the WHO South-East Asia Region committed to the objectives of *Decade for health workforce strengthening in the South-East Asia Region 2015–2024*, which identified improved rural retention as one of four key goals.^7^

To assess in more detail the health workforce retention challenges and country policy responses, in 2019 six country case studies (Bhutan, Chhattisgarh State in India, Indonesia, Myanmar, Sri Lanka and Thailand) were conducted. The case studies aimed at better understanding what rural retention policies had been implemented, how these policies were implemented and the impact of those interventions. The WHO 2010 framework was used to assess retention policy implementation.^4^

Findings showed that the six countries had implemented many of the 16 interventions of the WHO 2010 guidelines,^4^ usually as interlinked or bundled interventions. These countries most commonly used education strategies and regulatory interventions, but examples of financial incentives and personal and professional support were also found.^9^

Unfortunately, countries usually implemented retention policies without collecting baseline information for tracking progress and for rigorous assessment of outcomes. Moreover, monitoring, which could have informed policy adjustment, was reportedly lacking.

In these countries, the use of either routine statistics or special surveys on human resources for health outputs, outcome and impact of retention policy implementation was very limited, and attributing causality of the few impact results obtained was difficult.

However, a few examples of evaluation were available. Thailand conducted primary surveys that enabled assessment of the impact of policy interventions related to the Collaborative Project to Increase the Production of Rural Doctors, which was based on a special recruitment track of medical students from rural areas.^9^ Graduates from this project had a similar success rate at the national license examination (compared to students on the regular national entrance examination track), and had a higher probability of fulfilling mandatory service and competency.^10^ In India, data from three districts in Chhattisgarh State highlighted an increase in the availability of doctors, specialists and nurses between 2009 and 2018, as well as higher uptake of both outpatient and inpatient health services. Data from Bhutan showed a reduced vacancy rate of health assistants at Basic Health Units to the very low level of 3.3% (20/609).^9^

Findings from the case studies reaffirmed the evaluation challenges linked to very limited human resources for health data at primary health-care level, and lack of adequate and system-wide information systems to monitor and assess the impact of rural retention policy interventions.^9^

## Data on human resources for health

Despite the efforts to improve national data on primary health-care providers in recent years, including the early phase of national health workforce accounts, some key challenges remain. First: high fragmentation of data systems, in particular limited interoperability across systems and incomplete data, limited data from private and nongovernmental health providers who play a major role in some countries, and limited disaggregated data by gender, age and public and private distributions of health-care workers. Second: weak governance and coordination mechanisms between health professional training institutes and public and private employers. Third: lack of standardization of tools and definition of indicators across systems and countries. Fourth: weak capacity at subnational level to manage and make best use of data for local planning. Fifth: poor accessibility, quality and use of data for policy decision-making.^11^ Most importantly, data disaggregation of cadres of health workforce between hospital and primary health-care sectors does not exist in many countries and systems.

Some of these limitations can be addressed if countries fully adopt national health workforce accounts, aimed at improving human resources for health information systems and data.^12^ While the percentage of health workforce in ambulatory health care (presumed to be primary health-care level facilities) is a distribution indicator in the national health workforce accounts, the system is not specifically designed for monitoring rural retention policies in underserved areas.

## The new WHO guidelines

New evidence on effective interventions will be included in the revised guidelines on rural retention. The updated guidelines represent an opportunity to include a rural retention monitoring and evaluation framework and rural retention indicators. This inclusion will guide countries in designing, implementing and assessing the impact of rural retention interventions and will emphasize the importance of strengthening human resources for health information systems in primary health care to be able to monitor and document impact of policy interventions.

## Assessing rural retention policies

In countries with limited data and health information system capacity, we propose simplified and prioritized indicators to measure the process, outputs and outcomes of retention policy interventions, based on an existing framework^6^ and from experience in low- and middle-income countries in the South-East Asia Region (Table 1). The approach can use routine administrative data such as employment databases and patient satisfaction interviews, or use surveys of health professional students who are about to graduate, or of in-service primary health-care staff members. Most importantly, capacities to analyse and use this evidence to improve implementation of rural retention strategies should exist.

**Table 1:** Proposed assessment indicators for rural retention and approaches

Dimension of assessment	Indicators	Approaches
**Policy implementation**		
Implementation of the 16 global policy recommendations on “increasing access to health workers in remote and rural areas through improved retention”	Level of implementation: qualitative assessment or Likert scale survey	Regular internal assessment by health ministries and key partners aim to identify policy, implementation and resources gap, and the comprehensiveness of bundle interventions
**Output**		
Intention to stay in primary health care, five-year retention rate, proportion of health workers remaining in rural area	Per cent intention to stay in primary health care. Five-year survival analysis using Kaplan–Meier survival curves	Regular surveys of (i) different cadres of new graduate, (ii) in-service primary health-care workforce, on five-year intention to stay and leave the country, stay in primary health care, and postgraduate training need
Job satisfaction	Average satisfaction score by different cadres; key push factors	Primary health-care workforce survey of job satisfaction, identify and fix the gaps in particular on remunerations, workplace safety, professional isolation and support
No. and cadre mix of health workers recruited in primary health care, duration of service, percentage of filled post and vacancy rate	Trend of filled post and vacancy rate, by key cadres of professional, average year of primary health-care services	Strengthen human resources for health information that capture employment, mobility by professional mix
Health workforce absenteeism	Trend of absenteeism rate and reasons	Unannounced supervisory visits and assessment of reasons of absenteeism
Retention stability	Percentage of health workers who stay after one, two or three years	Routine employment database
Staff turnover	Percentage of staff remaining after one year	Routine employment database
**Outcome**		
Health workforce performance: availability, competence, productivity, responsiveness	Level of competency (clinical, public health, communication and cultural), workload per full-time equivalent staff	Regular surveys that assess the competency, productivity and responsiveness among in-service primary health-care workforce
Coverage of key health interventions and health service use by population	Coverage of essential maternal, newborn, child and adolescent services, noncommunicable disease screening and treatment	Strengthen routine health information systems that capture coverage of immunization, antenatal care, delivery by skilled birth attendants, and others such as noncommunicable disease services
Primary health-care responsiveness	Health systems responsiveness score	Household survey to assess the primary health-care responsiveness to citizens’ health needs
Patient satisfaction	Average score of patient satisfaction with different services	Patient exit self-administered or interview questionnaire survey

## The way forward

Improved retention of health workers in rural and underserved areas is fundamental for a well performing primary health-care system that contributes to achieving UHC and enhancing equity.

Countries in the South-East Asia Region are implementing many of the rural retention interventions proposed in the 2010 WHO rural retention guidelines.^4^ However, these countries are generally not monitoring the outcome, effectiveness and impact of these interventions. The underlying reasons for this gap are the weak human resources for health information systems and limited evaluation and research capacity.

Countries need to strengthen their human resources for health information systems, in particular to monitor progress of retention at primary health-care level. Countries also need to develop and apply standardized rural retention indicators that can inform policy-makers and managers and enable evidence-informed decisions that improve the deployment and retention of health workers in rural and underserved areas.

The updated WHO rural retention guidelines should be supported by a monitoring and evaluation framework that includes relevant and applicable indicators. The guidelines should also emphasize the importance of strengthening the human resources for health information systems and use these systems to guide policy implementation in primary health care.
